# Cell response analysis in SARS-CoV-2 infected bronchial organoids

**DOI:** 10.1038/s42003-022-03499-2

**Published:** 2022-05-30

**Authors:** Emi Sano, Tatsuya Suzuki, Rina Hashimoto, Yumi Itoh, Ayaka Sakamoto, Yusuke Sakai, Akatsuki Saito, Daisuke Okuzaki, Daisuke Motooka, Yukiko Muramoto, Takeshi Noda, Tomohiko Takasaki, Jun-Ichi Sakuragi, Shohei Minami, Takeshi Kobayashi, Takuya Yamamoto, Yasufumi Matsumura, Miki Nagao, Toru Okamoto, Kazuo Takayama

**Affiliations:** 1 Center for iPS Cell Research and Application (CiRA), Kyoto University, Kyoto, 606-8507 Japan; 2 Institute for Advanced Co-Creation Studies, Research Institute for Microbial Diseases, Osaka University, Suita, 565-0871 Japan; 3 Laboratory of Veterinary Pathology, Joint Faculty of Veterinary Medicine, Yamaguchi University, Yamaguchi, 753-8511 Japan; 4 Department of Veterinary Science, Faculty of Agriculture, University of Miyazaki, Miyazaki, 889-2192 Japan; 5 Genome Information Research Center, Research Institute for Microbial Diseases, Osaka University, Suita, 565-0871 Japan; 6 Single Cell Genomics, Human Immunology, WPI Immunology Frontier Research Center, Osaka University, Suita, 565-0871 Japan; 7 Institute for Open and Transdisciplinary Research Initiatives, Osaka University, Suita, 565-0871 Japan; 8 Laboratory of Ultrastructural Virology, Institute for Frontier Life and Medical Sciences, Kyoto University, Kyoto, 606-8507 Japan; 9 Kanagawa Prefectural Institute of Public Health, Chigasaki, Kanagawa 253-0087 Japan; 10 Laboratory of Viral Replication, International Research Center for Infectious Diseases, Research Institute for Microbial Diseases, Osaka University, Suita, Osaka, 565-0871 Japan; 11 Institute for the Advanced Study of Human Biology (WPI-ASHBi), Kyoto University, Kyoto, 606-8501 Japan; 12 Medical-risk Avoidance based on iPS Cells Team, RIKEN Center for Advanced Intelligence Project (AIP), Kyoto, 606-8507 Japan; 13 AMED-CREST, Japan Agency for Medical Research and Development (AMED), Tokyo, 100-0004 Japan; 14 Department of Clinical Laboratory Medicine, Graduate School of Medicine, Kyoto University, Kyoto, 606-8303 Japan

**Keywords:** Respiratory tract diseases, SARS-CoV-2

## Abstract

The development of an in vitro cell model that can be used to study severe acute respiratory syndrome coronavirus 2 (SARS-CoV-2) research is expected. Here we conducted infection experiments in bronchial organoids (BO) and an BO-derived air-liquid interface model (BO-ALI) using 8 SARS-CoV-2 variants. The infection efficiency in BO-ALI was more than 1,000 times higher than that in BO. Among the bronchial epithelial cells, we found that ciliated cells were infected with the virus, but basal cells were not. Ciliated cells died 7 days after the viral infection, but basal cells survived after the viral infection and differentiated into ciliated cells. Fibroblast growth factor 10 signaling was essential for this differentiation. These results indicate that BO and BO-ALI may be used not only to evaluate the cell response to SARS-CoV-2 and coronavirus disease 2019 (COVID-19) therapeutic agents, but also for airway regeneration studies.

## Abstract (web summary)

Bronchial organoids (BO) and BO-derived air-liquid interface models (BO-ALI) are used to model SARS-CoV-2 infection of human bronchial epithelial cells, reporting that ciliated cells were permissive to the virus, unlike basal cells.

## Introduction

Coronavirus disease 2019 (COVID-19) was first reported in China in December 2019^1^ and declared a pandemic by the World Health Organization (WHO) in March 2020^2^. Severe pneumonia is most frequently observed in COVID-19 patients, and the number of COVID-19 patients and deaths are still increasing. These conditions have made it difficult for research on severe acute respiratory syndrome coronavirus 2 (SARS-CoV-2), which is the causative virus of COVID-19, to keep pace. SARS-CoV-2 is composed of four proteins: S (spike), E (envelope), M (membrane), and N (nucleocapsid) proteins. It is known that angiotensin-converting enzyme 2 (ACE2) is a SARS-CoV-2 receptor, and transmembrane serine proteinase 2 (TMPRSS2) is essential for priming S protein^3^. Thus, to accelerate SARS-CoV-2 research, a lung model that reproduces the viral life cycle with intact expression of these host factors is indispensable.

A number of animal and cell models that can be used for SARS-CoV-2 research have been reported^4^, but an in vitro lung model that can evaluate candidate therapeutic agents for COVID-19 is essential for conducting large-scale drug screening. Human airway and alveolar organoids are excellent tools that can faithfully mimic the lung functions of living organisms^5–8^. Therefore, bronchial organoids (BO), containing transient secretory, goblet, and ciliated cells, and alveolar organoids, containing type I and II alveolar epithelial cells, are widely used for SARS-CoV-2 research^9^. However, even if SARS-CoV-2 is added into the medium, it can only infect organoids from the basement membrane side. Using organoids-derived air-liquid interface cell-culture models (ALI), virus infection from the luminal side can be reproduced. Accordingly, in this study, we used organoid-derived ALI to reproduce virus infection from the luminal side.

It is known that severe acute bronchopneumonia is frequently observed in COVID-19 patients^10^. Thus, clarifying the mechanism by which the bronchial epithelial layer is destroyed and regenerated in the infected bronchi would contribute to new therapeutic agents for severe acute bronchopneumonia. Bronchial basal cells have the ability to differentiate into all of the major bronchial epithelial cells, including basal cells themselves, club, goblet, and ciliated cells. Cytokeratin 5 (KRT5), nerve growth factor receptor (NGFR), and transcription factor p63 (TR63) are known as markers for basal cells^11^. It has been reported that basal cells proliferate after the influenza infection^12,13^. It is also known that fibroblast growth factor receptor 2 (FGFR2) is required for the maintenance and differentiation of basal cells^14^. FGF10 is known to promote lung epithelial regeneration after lung injury^15^. The FGF10 signal has been reported to be downregulated in human lung diseases including bronchopulmonary dysplasia (BPD), idiopathic pulmonary fibrosis (IPF), and chronic obstructive pulmonary disease (COPD)^15^. However, the behavior of basal cells after SARS-CoV-2 infection and the effect of FGF signals on the cells have not been clarified. In this study, we tried to clarify and control the role of basal cells in COVID-19.

## Results

### Bronchial organoids were hardly infected with SARS-CoV-2

**Fig. 1:**
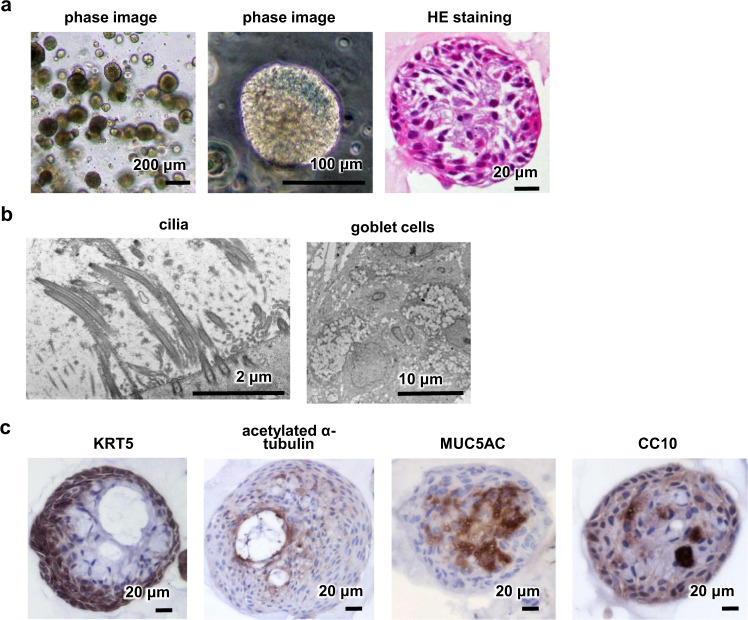
Generation of BO. **a** Phase and HE staining images of human bronchial organoids (BO). **b** TEM images of BO. Larger images are shown in Supplementary Fig. 1. **c** Immunohistochemistry analysis of KRT5 (basal cell marker), acetylated α-tubulin (ciliated cell marker), MUC5AC (goblet cell marker), and CC10 (club cell marker) in BO. Panels **a**–**c** are representative of three independent experiments.

We expanded and differentiated BO from normal human bronchial epithelial cells (NHBE) using expansion and differentiation media (Supplementary Table 1). Most NHBE were positive for KRT5, but negative for acetylated α-tubulin, suggesting that most NHBE were basal cells (Supplementary Fig. 1a). Approximately 100 BO were present in 50 μL of Matrigel, and the diameter of each BO was around 100–200 μm (Fig. 1a). Transmission electron microscopy (TEM) images showed the presence of cilia and goblet cells (Fig. 1b and Supplementary Fig. 1b). Because the bronchi are composed of basal, ciliated, goblet, and club cells, immunohistochemical analysis of markers specific to these four cell types was performed. BO were also positive for KRT5, acetylated α-tubulin, MUC5AC, and CC10 (Fig. 1c). Based on these observations, we succeeded in generating BO from NHBE.

**Fig. 2:**
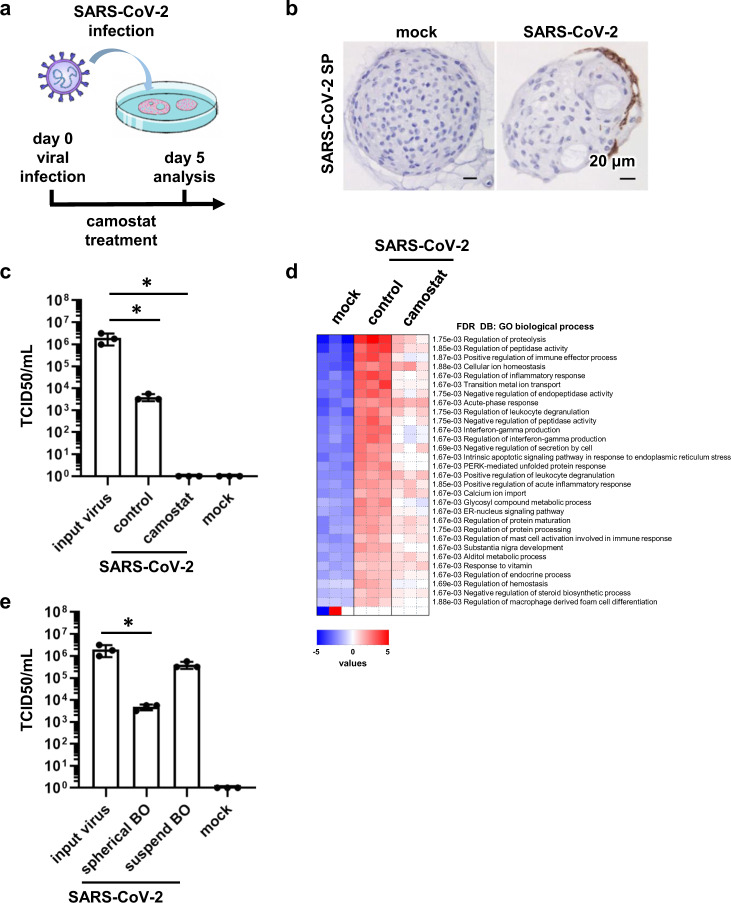
SARS-CoV-2 infection experiments in BO. **a** BO were infected with SARS-CoV-2 (1.3 × 10^5^ TCID50/well) in the presence or absence of 10 μM camostat and then cultured with differentiation medium for 5 days. **b** Immunohistochemistry analysis of SARS-CoV-2 Spike protein (SP) in the infected BO. **b** is representative of three independent experiments. **c** The amount of infectious virus in the supernatant was measured using the TCID50 assay. **d** Parametric Gene Set Enrichment Analysis (PGSEA) applied to GO biological process gene sets was performed in uninfected BO (mock), infected BO (control), and infected BO treated with 10 μM camostat (camostat). Three technical repeats were performed per sample for the RNA-seq analysis. **e** The amount of infectious virus in the supernatant of spherical and suspended BO was measured using the TCID50 assay. In panels **c** and **e**, statistical significance was evaluated by one-way analysis of variance (ANOVA) followed by Tukey’s post hoc tests (*P < 0.05). In panels **c** and **e**, data represent mean ± SD from three independent experiments.

Next, BO were infected with SARS-CoV-2 and then cultured in differentiation medium for 5 days (Fig. 2a). The virus contained in the medium infected BO from the basement membrane side. Immunohistochemical analysis showed that SARS-CoV-2 S protein (SP)-positive cells were rarely observed in BO and only in a part of the outer edge (Fig. 2b and Supplementary Fig. 2a). Consistently, the infectious virus was slightly detected in infected BO, and its production was decreased by treatment with camostat (Fig. 2c). The accumulation of lactate dehydrogenase (LDH) was not observed in the culture medium of infected BO (Supplementary Fig. 2b), suggesting that cytotoxicity was not caused by the infection. After the infection, qPCR and RNA-seq analyses showed that the expression levels of innate immune response-related genes were slightly enhanced (Fig. 2d and Supplementary Figs. 2c, 3). These results suggest that the SARS-CoV-2 rarely infects spherical BO.

To improve the infection efficiency in BO, the contact time of BO with SARS-CoV-2 was lengthened, but the infection efficiency did not change (Supplementary Fig. 2d). However, the infection efficiency in suspended BO was significantly higher than that in spherical BO (Fig. 2e). Immunofluorescence analysis in suspended BO treated with SARS-CoV-2 demonstrated that SARS-CoV-2 infects acetylated α-tubulin-positive ciliated cells but not KRT5-positive basal cells (Supplementary Fig. 4a, b). This effect might be because ciliated cells, not basal cells, express ACE2, which is a receptor for SARS-CoV-2 (Supplementary Fig. 4c). These results suggest that the infection efficiency of spherical BO is quite low because SARS-CoV-2 cannot access ciliated cells located in the BO lumen.

### Bronchial organoids-derived air–liquid interface cell-culture models were efficiently infected with SARS-CoV-2

**Fig. 3:**
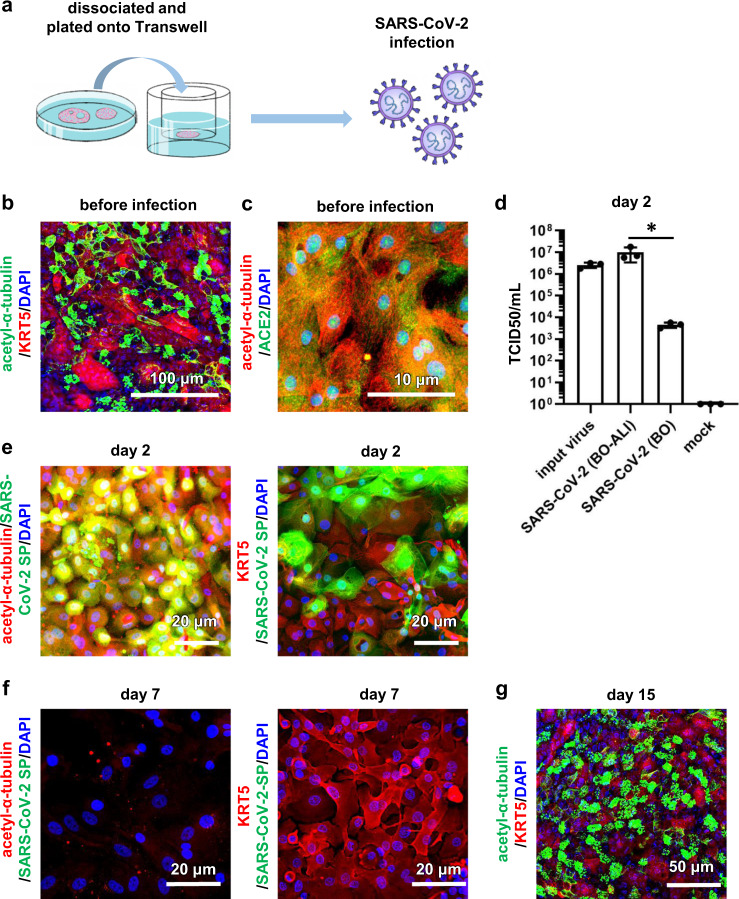
SARS-CoV-2 infection experiments in BO-ALI. **a** BO-ALI were infected with SARS-CoV-2 (1.3 × 10^5^ TCID50/well) and then cultured with differentiation medium for 2 days. **b** Immunofluorescence analysis of KRT5 (red) and acetylated α-tubulin (green) in uninfected BO-ALI. Nuclei were counterstained with DAPI (blue). **c** Immunofluorescence analysis of ACE2 (green) and acetylated α-tubulin (red) in uninfected BO-ALI. Nuclei were counterstained with DAPI (blue). **d** The amount of infectious virus in the supernatant of infected BO or BO-ALI was measured by the TCID50 assay. Statistical significance was evaluated using one-way ANOVA followed by Tukey’s post hoc test (*P < 0.05). Data represent the mean ± SD from three independent experiments. **e** Immunofluorescence analysis of SARS-CoV-2 Spike protein (SP) (green) and acetylated α-tubulin (red) in uninfected BO-ALI 2 days after the infection. Immunofluorescence analysis of SARS-CoV-2 SP (green) and KRT5 (red) in uninfected BO-ALI. Nuclei were counterstained with DAPI (blue) 2 days after the infection. **f** Immunofluorescence analysis of SARS-CoV-2 SP (green) and acetylated α-tubulin (red) in infected BO-ALI 7 days after the infection. Immunofluorescence analysis of SARS-CoV-2 SP (green) and KRT5 (red) in infected BO-ALI 7 days after the infection. Nuclei were counterstained with DAPI (blue). **g** Immunofluorescence analysis of KRT5 (red) and acetylated α-tubulin (green) in BO-ALI 15 days after the infection. Nuclei were counterstained with DAPI (blue). Panels **b**, **c**, **e**–**g** are representative of three independent experiments.

BO-ALI were used to mimic viral infection from the luminal side of bronchi. Expanded BO were seeded in Transwell inserts and cultured in differentiation medium (Fig. 3a). Immunofluorescence analysis showed that acetylated α-tubulin- and KRT5-positive cells were observed in BO-ALI (Fig. 3b) and that ACE2 colocalized with α-tubulin (Fig. 3c). These data suggest that BO-ALI contain ciliated cells that strongly express viral receptors. Note that there was no difference in the gene expression level of SARS-CoV-2-related markers or bronchial epithelial cell markers between BO and BO-ALI (Supplementary Fig. 5a, b). In addition, there was no difference in protein expression and localization of ACE2 and TMPRSS2 between BO and BO-ALI (Supplementary Fig. 5c). Next, BO and BO-ALI were infected with SARS-CoV-2 B and B.1.1.214, and then cultured in differentiation medium for 2 days (Fig. 3d and Supplementary Fig. 6a, respectively). The production of infectious virus in BO-ALI was significantly higher than in BO. The production of infectious virus in BO-ALI (1.00 × 10^7^ TCID50/mL) was higher than that of suspended BO (3.98 × 10^5^ TCID50/mL) (Figs. 2e and 3d), suggesting that the difference in infection efficiency between BO-ALI and BO was not due to Matrigel embedding. Immunofluorescence analysis showed that SARS-CoV-2 SP colocalized with acetylated α-tubulin, but not with KRT5 (Fig. 3e). These results suggest that ciliated cells in BO-ALI were efficiently infected with SARS-CoV-2. At 7 days after the infection, acetylated α-tubulin- and SARS-CoV-2 SP-positive cells were not observed (Fig. 3f), suggesting that the ciliated cells died due to the viral infection. Immunofluorescence analysis showed that the death was due to apoptosis by day 4 after the infection (Supplementary Fig. 6b). On the other hand, KRT5-positive basal cells survived 7 days after the infection (Fig. 3f). Fifteen days after the infection, the surviving basal cells differentiated into acetylated α-tubulin-positive ciliated cells, forming a bronchial epithelial layer (Fig. 3g). We also confirmed that infectious virus was not present in the cell-culture supernatant 7 and 15 days after the infection (Supplementary Fig. 6c). These observations suggest that basal cells play an important role in the repair of the bronchial epithelial layer after the virus infection.

### Evaluation of COVID-19 therapeutic agents using bronchial organoids-derived air–liquid interface cell-culture models

**Fig. 4:**
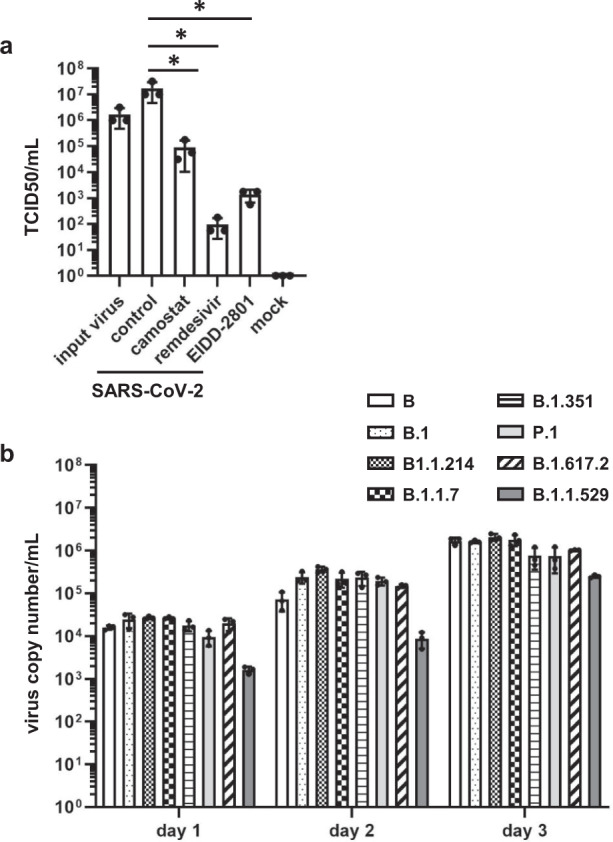
Evaluation of COVID-19 therapeutic drugs using BO-ALI. **a** BO-ALI were infected with SARS-CoV-2 (1.3 × 10^5^ TCID50/well) in the presence or absence of 10 μM camostat, 1 μM remdesivir, or 1 μM EIDD-2801 and then cultured with differentiation medium for 2 days. The amount of infectious virus in the supernatant was measured using the TCID50 assay. Statistical significance was evaluated using one-way ANOVA followed by Tukey’s post hoc test (*P < 0.05). **b** BO-ALI were infected with SARS-CoV-2 B, B.1, B.1.1.214, B.1.1.7, B.1.351, P.1, B.1.617.2, or B.1.1.529 (1.3 × 10^5^ TCID50/well) and then cultured with differentiation medium. The virus copy number in the supernatant was measured by qPCR 1, 2, or 3 days after the infection. Data represent the mean ± SD from three independent experiments.

We next examined whether the antiviral effect of COVID-19 therapeutic agents can be evaluated using BO-ALI. In addition to camostat, the antiviral effects of two RNA-dependent RNA polymerase (RdRp) inhibitors, remdesivir^16^ and EIDD-2801^17^, were investigated. After culturing infected BO-ALI with a drug-containing medium, a TCID50 assay was performed. TCID50 values were significantly decreased by camostat, remdesivir, and EIDD-2801 treatment (Fig. 4a). We also confirmed that camostat treatment canceled the SARS-CoV-2 infection-mediated expression of innate immune response genes (Supplementary Fig. 7). When BO-ALI were treated with 8 SARS-CoV-2 variants, the virus genome copy number in the cell-culture supernatant was temporarily increased (Fig. 4b). Thus, BO-ALI can be used to evaluate the efficacy of COVID-19 therapeutic agents for many SARS-CoV-2 variants.

### FGF10 is essential for surviving basal cells to regenerate the bronchial epithelial cell layer

**Fig. 5:**
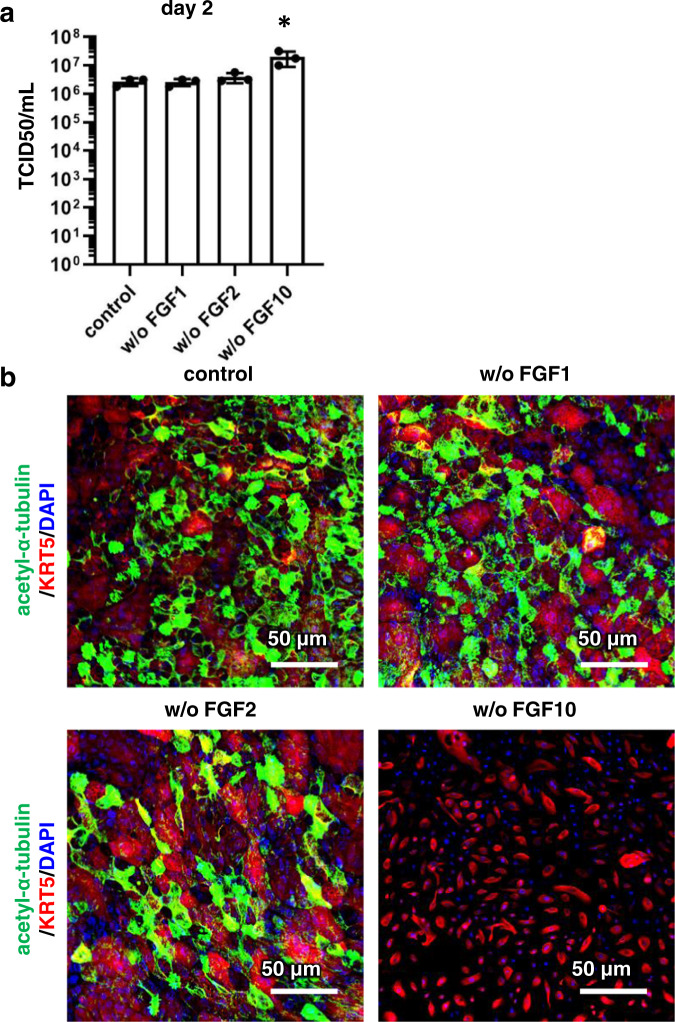
FGF10 is essential for the differentiation of basal cells. **a** BO-ALI were infected with SARS-CoV-2 (1.3 × 10^5^ TCID50/well) and then cultured with a differentiation medium for 2 (**a**) or 15 (**b**) days. After the infection, BO-ALI were cultured with differentiation medium with or without FGF1, 2, or 10. The amount of infectious virus in the supernatant was measured by the TCID50 assay 2 days after the infection. Statistical significance was evaluated by one-way ANOVA followed by Dunnett’s post hoc test (*P < 0.05). Data represent the mean ± SD from three independent experiments. **b** Immunofluorescence analysis of acetylated α-tubulin (green) and KRT5 (red) 15 days after the infection. Nuclei were counterstained with DAPI (blue). Panel **b** is representative of three independent experiments.

Because mouse FGF10 is known to be important for the replication of mouse basal cells^15^, we investigated the effect of human recombinant FGFs on viral infection and subsequent bronchial epithelial layer regeneration. The differentiation medium used in this experiment contained human recombinant FGF1, 2, and 10. By removing FGF10 from the medium, the production of the infectious virus in BO-ALI increased (Fig. 5a). The number of acetylated α-tubulin-positive ciliated cells was increased by culturing in medium without FGF10 (Supplementary Fig. 8), which may explain the increased infection efficiency in medium without FGF10. In addition, due to the absence of FGF10, surviving basal cells did not proliferate or differentiate into acetylated α-tubulin-positive ciliated cells (Fig. 5b). On the other hand, if FGF1 or 2 was removed from the differentiation medium, the surviving basal cells proliferated and could differentiate into acetylated α-tubulin-positive ciliated cells. These results suggest that FGF10 is essential for surviving basal cells to regenerate the bronchial epithelial cell layer.

**Fig. 6:**
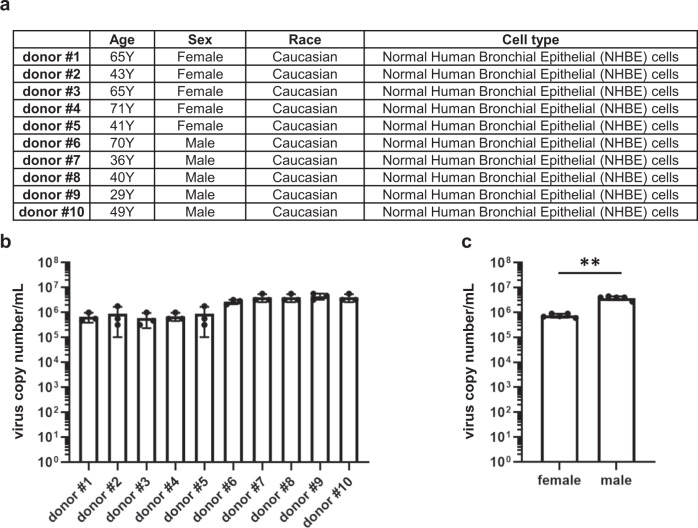
Infection experiments of BO-ALI generated from ten donors. **a** Donor information of the NHBE cells used in this study. **b**, **c** BO-ALI were infected with SARS-CoV-2 (1.3 × 10^5^ TCID50/well) and then cultured with differentiation medium for 2 days. The viral RNA copy number in the cell-culture supernatant was measured by qPCR. Statistical significance was evaluated using an unpaired Student’s *t* test (**P < 0.01). Data represent the mean ± SD from three independent experiments.

To investigate whether there is a difference in virus replication efficiency among donors, BO-ALI were generated from NHBE obtained from ten donors. The donor information is summarized in Fig. 6a. Notably, the production of infectious virus in male BO-ALI was higher than in female BO-ALI (Fig. 6b, c). Overall, our BO-ALI have the potential to reproduce individual differences, including gender differences, in the virus replication efficiency. It may also be possible to reproduce racial differences in virus replication efficiency by generating BO-ALI from NHBEs of different races.

## Discussion

In this study, we generated BO and BO-ALI and applied them to SARS-CoV-2 research. The incorporation of mechanical stress into our organoid system could improve the accuracy of SARS-CoV-2 research. The human airway is always exposed to shear stress due to airflow. It has been reported that a functional in vitro lung model can be generated using a device capable of medium perfusion and expansion/contraction (organs-on-a-chip)^18^. Recently, it was reported that the infection and replication of SARS-CoV-2 can be observed by culturing bronchial epithelial cells and pulmonary microvascular endothelial cells on a microfluidic device^19,20^. By applying our BO and BO-ALI to a similar microfluidic device, we may be able to construct an in vitro bronchi model that more closely mimics the living body.

In experiments using BO-ALI, we confirmed that basal cells survived after the viral infection, but also discovered that FGF10 is essential for their proliferation and differentiation (Figs. 3 and 5). Therefore, FGF10 may be effective at regenerating bronchi damaged by viral infection. In addition, the transplantation of basal cells may promote the regeneration of bronchi damaged by the viral infection. In vivo SARS-CoV-2 models such as cynomolgus monkeys, hamsters, and ferrets would clarify these possibilities^4,21,22^. Although we confirmed that FGF10 is required for the growth and differentiation of basal cells, the key FGFR in basal cells has not been identified. FGFR1b and 2b are known receptors for FGF10^23^, and their expression levels and functions in basal cells should be explored. Furthermore, because FGF3, FGF7, and FGF22 are in the same subfamily as FGF10^23^, they too could contribute to the regeneration of the bronchial epithelial layer.

We showed that ciliated cells, but not basal cells, are efficiently infected with SARS-CoV-2 (Fig. 3e and Supplementary Fig. 4). However, we did not analyze club or goblet cells. Future research should clarify whether these secretory cells are infected with SARS-CoV-2. Because club cells have the potential to dedifferentiate into basal cells^24^, they may contribute to the regeneration of the bronchial epithelial layer.

BO-ALI was used to reproduce the destruction and regeneration but not the bronchial inflammation of the infected bronchial epithelial layer. To reproduce tissue inflammation in vitro, it is essential to develop a model containing immune cells. Currently, antiviral and anti-inflammatory drugs are being developed to treat COVID-19, but few drugs are being developed to promote the regeneration of damaged tissue. Although this study focused on bronchi, it may be possible to identify cells and factors that promote damaged tissue regeneration in other organs using a similar approach.

In conclusion, we verified the usefulness of BO and BO-ALI for the study of SARS-CoV-2 study. Because spherical BO can be expanded, they are also useful as a cellular resource for drug screening. However, their infection efficiency is low, because only the outer layer of the BO contacts SARS-CoV-2 (Fig. 2). Therefore, suspended BO or BO-ALI are preferred for infection experiments and evaluation of COVID-19 therapeutic agents. Recently, a technique for generating inside-out organoids has been developed^25^. This technique could increase the efficiency of infection experiments even if using spherical BO. Following these findings, we expect BO and BO-ALI will accelerate pharmaceutical research for infectious diseases, including COVID-19.

## Methods

### BO and BO-ALI culture

Normal human bronchial epithelial cells (NHBE, #CC-2540, Lonza) were used as human bronchial basal cells in this study. To generate BO, NHBE were suspended in 10 mg/mL cold Matrigel growth factor reduced (GFR) basement membrane matrix. 50 μL drops of cell suspension were solidified on pre-warmed Nunc cell-culture treated multidishes (24-well plates) at 37 °C for 10 min, and then 500 μL of expansion medium (the composition is shown in Supplementary Table 1) was added to each well. Expansion medium was changed every 2 days. For passaging, BO were suspended in 1 mL of 0.5 mM EDTA/PBS (Nacalai tesque) and mechanically sheared using a P1000 pipette tip. Then, 2 mL TrypLE Select (Thermo Fisher Scientific) was added to the suspension. After incubating for 5 min at room temperature, the BO were again mechanically sheared using the P1000 pipette tip. In all, 7 mL of expansion medium was added, and the organoid suspension tubes were centrifuged at 400 rpm. Organoid fragments were re-suspended in cold expansion medium and seeded as above. BO were passaged every 10 days. To mature the BO, expanded BO were cultured with differentiation medium (the composition is shown in Supplementary Table 1) for 5 days. BO can be cryopreserved using STEM-CELLBANKER GMP grade (TaKaRa Bio). To generate BO-ALI, expanding BO cultured in a 24-well plate were seeded into Transwell inserts (Corning) in a 24-well plate. To promote their maturation, BO-ALI were cultured with differentiation medium for 5 days.

### SARS-CoV-2 preparation

The SARS-CoV-2 strains B.1.1.214 (GISAID accession number: EPI_ISL_2897162) and B.1.617.2 (GISAID accession number: EPI_ISL_9636792) were isolated from a nasopharyngeal swab sample of a COVID-19 patient. This study has been approved by the research ethics committee of Kyoto University. SARS-CoV-2 B (SARS-CoV-2/Hu/DP/Kng/19-020 and SARS-CoV-2/Hu/DP/Kng/19-027) were provided by the Kanagawa Prefectural Institute of Public Health, SARS-CoV-2 B.1 (NR-53514) and SARS-CoV-2 B.1.1529 (NR-56461) were obtained from BEI Resources, and SARS-CoV-2 B.1.1.7, B.1.351, and P.1 were provided by the National Institute of Infectious Diseases. SARS-CoV-2 B was used for all figures other than Fig. 4b and Supplementary Fig. 6a. The virus was plaque-purified and propagated in Vero cells. SARS-CoV-2 was stored at −80 °C. Note that we confirmed that there is no change in the sequence of furin cleavage site even after the viral replication in Vero cells. However, repeated virus passages increase the risk of acquiring in vitro-specific mutations. Thus, we performed virus passages less than 3 times after the SARS-CoV-2 was isolated or obtained. All experiments including virus infections were done in biosafety level 3 facilities at Kyoto University and Osaka University strictly following regulations.

### SARS-CoV-2 infection and drug treatment

BO cultured in a 24-well plate (~100 organoids) were infected with 1.3 × 10^5^ TCID50 of SARS-CoV-2. In the BO infection experiments, one-half of the differentiation medium containing SARS-CoV-2 was replaced with fresh differentiation medium every day. In the drug treatment experiments, the infected BO were cultured with differentiation medium containing camostat (SML0057, Sigma-Aldrich) for 5 days. When performing the TCID50 assay, differentiation medium was replaced with fresh medium 24 h before the cell-culture supernatant was collected.

In the BO-ALI infection experiments, BO-ALI cultured in Transwell inserts in a 24-well plate (generated from approximately 100 organoids) were infected with 1.3 × 10^5^ TCID50 of SARS-CoV-2 for 90 min. Medium containing SARS-CoV-2 was then replaced with fresh differentiation medium. In the drug treatment experiments, the infected BO-ALI were cultured with differentiation medium containing camostat, remdesivir (A17170, Clinisciences), or EIDD-2801 (HY-135853, MedChemExpress) for 2 days. When performing the TCID50 assay, differentiation medium was replaced with a fresh medium 24 h before the cell-culture supernatant was collected.

### Viral titration of SARS-CoV-2

Viral titers were measured using median tissue culture infectious dose (TCID50) assays at a Biosafety Level 3 laboratory (Kyoto University). TMPRSS2/Vero cells (JCRB1818, JCRB Cell Bank)^26^, which were cultured with Minimum Essential Media (MEM, Sigma-Aldrich) supplemented with 5% fetal bovine serum (FBS), and 1% penicillin/streptomycin were seeded into 96-well cell-culture plates (Thermo Fisher Scientific). The samples were serially diluted tenfold from 10^−1^ to 10^−8^ in cell-culture medium. Dilutions were placed onto TMPRSS2/Vero cells in triplicate and incubated at 37 °C for 96 h. Cytopathic effects were evaluated under a microscope. TCID50/mL was calculated using the Reed-Muench method.

### Quantitative PCR

Total RNA was isolated from BO, BO-ALI, and bronchial basal cells using ISOGENE II (NIPPON GENE). cDNA was synthesized using 500 ng of total RNA with a Superscript VILO cDNA synthesis kit (Thermo Fisher Scientific). Real-time RT-PCR was performed with the SYBR Green PCR Master Mix (Thermo Fisher Scientific) using a StepOnePlus real-time PCR system (Thermo Fisher Scientific). The relative quantitation of target mRNA levels was performed using the 2^-ΔΔCT^ method. The values were normalized by those of the housekeeping gene, *glyceraldehyde 3-phosphate dehydrogenase* (*GAPDH*). The PCR primer sequences are shown in Supplementary Table 2.

### Quantification of viral RNA copy number

The cell-culture supernatant was mixed with an equal volume of 2× RNA lysis buffer (distilled water containing 0.4 U/μL SUPERase•In Rnase Inhibitor (Thermo Fisher Scientific), 2% Triton X-100, 50 mM KCl, 100 mM Tris-HCl (pH 7.4), and 40% glycerol) and incubated at room temperature for 10 min. The mixture was diluted 10 times with distilled water. Viral RNA was quantified using a One Step TB Green PrimeScript PLUS RT-PCR Kit (Perfect Real Time) (Takara Bio) on a QuantStudio 1 Real-Time PCR System (Thermo Fisher Scientific). The primers used in this experiment are shown in Supplementary Table 2. Standard curves were prepared using SARS-CoV-2 RNA (10^5^ copies/μL) purchased from Nihon Gene Research Laboratories.

### Ultrathin section transmission electron microscopy (TEM)

BO were fixed in phosphate-buffered 2% glutaraldehyde and subsequently post-fixed in 2% osmium tetraoxide for 2 h at 4 °C. After fixation, they were dehydrated in a graded series of ethanol and embedded in epoxy resin. Ultrathin sections were cut, stained with uranyl acetate and lead staining solution, and examined using an electron microscope (HITACHI H-7600) at 100 kV.

### Histopathology and immunofluorescence

Fixed BO samples were processed and embedded in paraffin. Then they were cut into 2-μm-thick sections. The sections were deparaffinized, rehydrated, and stained with hematoxylin and eosin (HE). The sections were then examined using a microscope (BX53 microscope with DP73 camera, Olympus Corporation).

For the immunohistochemical stain assay, the formalin-fixed and paraffin-embedded BO samples were treated with pH 6.0 citrate buffer for 30 s at 125 °C in a pressure cooker (Dako Japan) as antigen retrieval. Sections were incubated with each antibody (Supplementary Table 3), followed by Histofine Simple Stain MAX-PO (Nichirei Biosciences). The sections were visualized using a Peroxidase Stain DAB Kit (Nacalai Tesque) before counterstaining with Meyer’s hematoxylin.

For the double-immunofluorescence staining assay of infected BO, the sections were deparaffinized and subjected to antigen retrieval by treating them with 0.5% trypsin for 30 min. Then the sections were blocked by 5% skim milk with albumin obtained from Bovine Serum Cohn Fraction V, pH 7.0 (Wako Pure Chemical Industries), in PBS for 30 min at room temperature to avoid non-specific reactions. The sections were then incubated with a primary antibody (Supplementary Table 3) overnight at 4 °C, washed, and incubated with a secondary antibody (Supplementary Table 3) for 1 h at room temperature.

For the double-immunofluorescence staining assay of uninfected and infected ALI-BO, the cells were fixed with 4% paraformaldehyde in PBS at 4 °C. After blocking the cells with PBS containing 2% bovine serum albumin and 0.2% Triton X-100 at room temperature for 45 min, the cells were incubated with a primary antibody at 4 °C overnight and then with a secondary antibody at room temperature for 1 h. All antibodies used in this report are described in Supplementary Table 3.

### RNA-seq

Total RNA was prepared using the RNeasy Mini Kit (Qiagen). RNA integrity was assessed with a 2100 Bioanalyzer (Agilent Technologies). The library preparation was performed using a NEBNext Ultra II Directional RNA Library Prep Kit for Illumina (NEB) or a TruSeq stranded mRNA sample prep kit (Illumina) according to the manufacturer’s instructions. Sequencing was performed on an Illumina NextSeq500 or NovaSeq6000 platform in 152- or 101-base single-end mode, respectively. Fastq files were generated using bcl2fastq2. Adapter sequences were trimmed from the raw reads by cutadapt ver 2.7. The trimmed reads were mapped to the human reference genome sequences (hg19) using HISAT2 ver 2.1.0. The raw counts were calculated using featureCounts ver 2.0.0 and used for heatmap visualization with integrated differential expression and pathway analysis (iDEP, http://ge-lab.org/idep/)^27^. Raw data concerning this study were submitted under Gene Expression Omnibus (GEO) accession number GSE150819.

### LDH assay

After the SARS-CoV-2 infection, the release of LDH was monitored from an aliquot of 250 μL supernatant using the LDH-Glo cytotoxicity assay (Promega) according to the manufacturer’s instructions. The absorbance was determined with a Bio-Rad microplate reader (Bio-Rad, US) at wavelength 490 nm. The release of LDH in uninfected cells was used as a control.

### Statistics and reproducibility

Statistical significance was evaluated by unpaired Student’s *t* test or one-way analysis of variance (ANOVA) followed by Tukey’s or Dunnett’s post hoc tests. Details are described in the figure legends. Reproducibility was confirmed by performing three independent replicates as described in the figure legend.

### Reporting summary

Further information on research design is available in the Nature Research Reporting Summary linked to this article.

## Supplementary information

Supplementary Information

Description of Additional Supplementary Files

Supplementary Data 1

Reporting Summary

## Data Availability

RNA-seq data were submitted under Gene Expression Omnibus (GEO) accession number GSE150819. All source data underlying the graphs and charts are uploaded as Supplementary Data in Excel format. All other data are available from the corresponding authors on reasonable request. All unique/stable reagents generated in this study are available from the corresponding authors with a completed Materials Transfer Agreement.
