# Supplementary material for: Cell response analysis in SARS-CoV-2 infected bronchial organoids
Source: Commun Biol. 2022 May 30;5:516. doi: 10.1038/s42003-022-03499-2 (PMC9151746; doi:10.1038/s42003-022-03499-2)
Supplement: Supplementary file 1 — Supplementary Information [file 42003_2022_3499_MOESM1_ESM.pdf]

Supplemental manuscript

Title

Cell response analysis in SARS-CoV-2 infected bronchial organoids

Authors and affiliations

Emi Sano[1,#], Tatsuya Suzuki[2,#], Rina Hashimoto[1,#], Yumi Itoh[2], Ayaka Sakamoto[1], Yusuke Sakai[3], Akatsuki Saito[4], Daisuke Okuzaki[5,6,7], Daisuke Motooka[5], Yukiko Muramoto[8], Takeshi Noda[8], Tomohiko Takasaki[9], Jun-Ichi Sakuragi[9], Shohei Minami[10], Takeshi Kobayashi[10], Takuya Yamamoto[1,11,12,13], Yasufumi Matsumura[14], Miki Nagao[14], Toru Okamoto[2*], Kazuo Takayama[1,13*]

[1] Center for iPS Cell Research and Application (CiRA), Kyoto University, Kyoto 606-8507, Japan

[2] Institute for Advanced Co-Creation Studies, Research Institute for Microbial Diseases, Osaka University, Suita 565-0871, Japan

[3] Laboratory of Veterinary Pathology, Joint Faculty of Veterinary Medicine, Yamaguchi University, Yamaguchi 753-8511, Japan

[4] Department of Veterinary Science, Faculty of Agriculture, University of Miyazaki, Miyazaki 889-2192, Japan

[5] Genome Information Research Center, Research Institute for Microbial Diseases, Osaka University, Suita 565-0871, Japan

[6] Single Cell Genomics, Human Immunology, WPI Immunology Frontier Research Center, Osaka University, Suita 565-0871, Japan

[7] Institute for Open and Transdisciplinary Research Initiatives, Osaka University, Suita 565-0871, Japan

[8] Laboratory of Ultrastructural Virology, Institute for Frontier Life and Medical Sciences, Kyoto University, Kyoto 606-8507, Japan

[9] Kanagawa Prefectural Institute of Public Health, Kanagawa 253-0087, Japan

[10] Laboratory of Viral Replication, International Research Center for Infectious Diseases, Research Institute for Microbial Diseases, Osaka University, Suita, Osaka, 565-0871 Japan

[11] Institute for the Advanced Study of Human Biology (WPI-ASHBi), Kyoto University, Kyoto 606-8501 Japan

[12] Medical-risk Avoidance based on iPS Cells Team, RIKEN Center for Advanced Intelligence Project (AIP), Kyoto 606-8507, Japan

[13] AMED-CREST, Japan Agency for Medical Research and Development (AMED), Tokyo 100-0004, Japan
[14] Department of Clinical Laboratory Medicine, Graduate School of Medicine, Kyoto University, Kyoto 606-8303, Japan
[#] These authors contributed equally.

***Corresponding authors**
Dr. Kazuo Takayama
Center for iPS Cell Research and Application (CiRA), Kyoto University, Shogoin Kawaharacho 53, Sakyo-ku, Kyoto 606-8507, Japan
Phone: +81-75-366-7362, FAX: +81-75-366-7074
E-mail: kazuo.takayama@cira.kyoto-u.ac.jp

Dr. Toru Okamoto
Institute for Advanced Co-Creation Studies, Research Institute for Microbial Diseases, Osaka University, Yamadaoka 3-1, Suita 565-0871, Japan
Phone: +81-6-6879-8330, FAX: +81-6-6879-8330
E-mail: toru@biken.osaka-u.ac.jp

Supplementary Figures

Supplementary Figure 1 Characterization of NHBE and BO

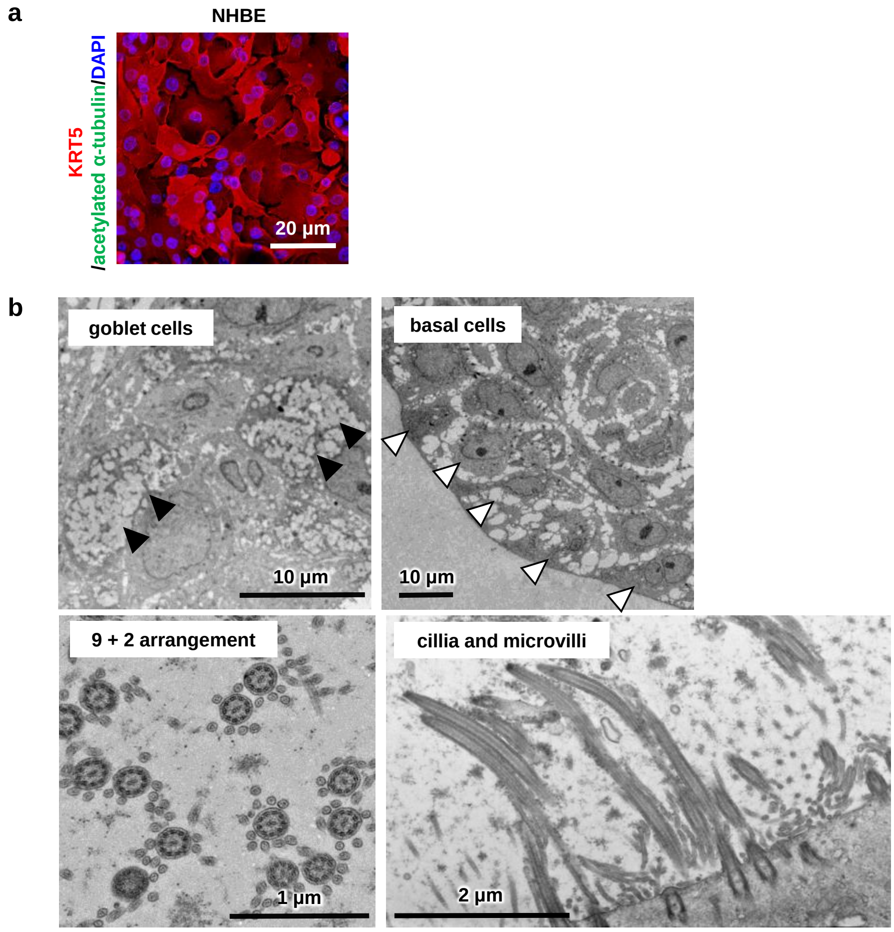

(**a**) Immunofluorescence analysis of acetylated α-tubulin (green) and KRT5 (red) in normal human bronchial epithelial cells (NHBE). Nuclei were counterstained with DAPI (blue). (**b**) TEM images of BO. Black arrows, goblet cells; white arrows, basal cells. Magnified TEM images from **figure 1b** are shown. Goblet cells, basal cells, 9+2 arrangement, cilia, and microvilli can be observed. **Supplementary Figures 1a and 1b** are representative of three independent experiments.

Supplementary Figure 2 Characterization of infected BO

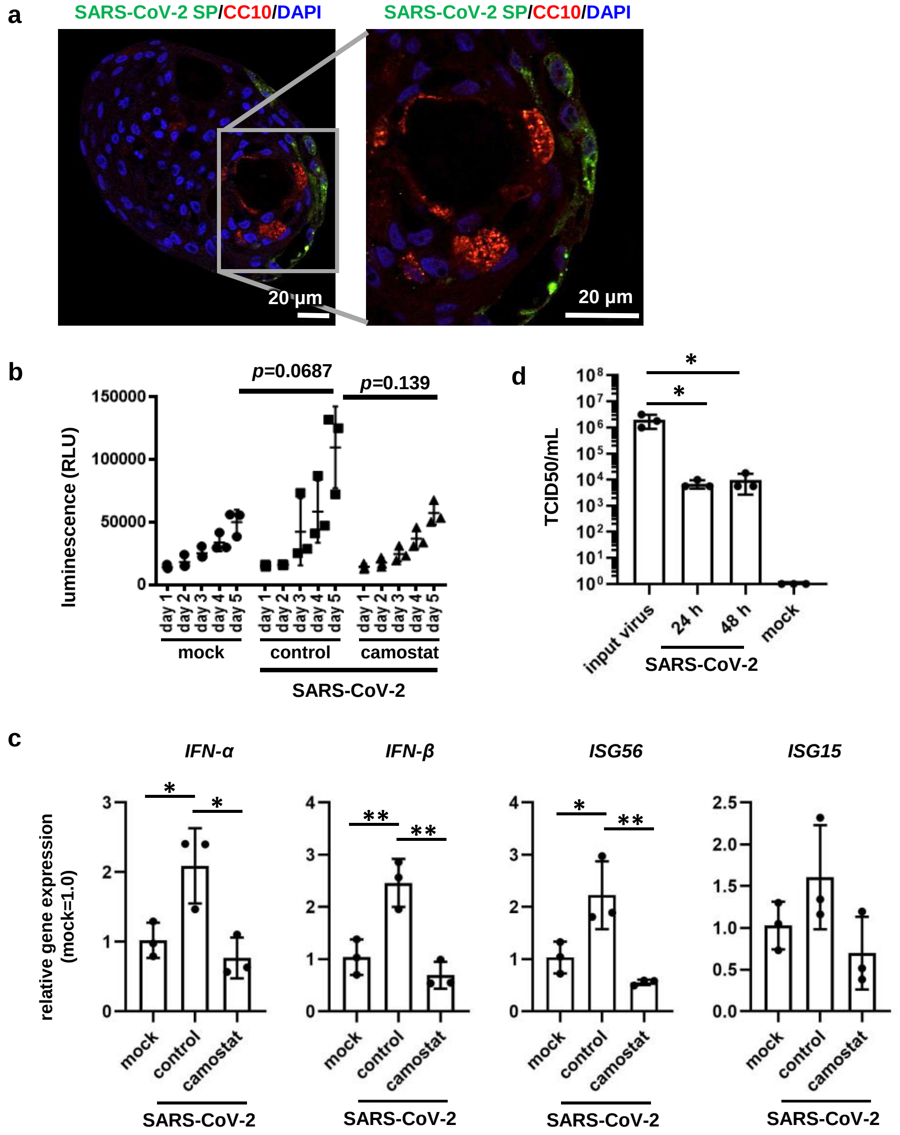

(**a**) Immunofluorescence analysis of SARS-CoV-2 SP (green) and CC10 (red) in infected BO. Nuclei were counterstained with DAPI (blue). (**b**) BO were infected with SARS-CoV-2 (1.3×10^{5} TCID50/well) in the presence or absence of 10 µM camostat and then cultured with differentiation medium for 5 days. At days 1, 2, 3, 4, and 5 after the infection, an LDH assay was performed. Statistical significance was evaluated by one-way ANOVA followed by Dunnett's post-hoc tests (compared with "infected BO (SARS-CoV-2)"). **Supplementary Figure 2b** is representative of three independent experiments and values are expressed as the mean ± SD (three technical repeats). (**c**) The gene expression levels of *IFN-α*, *IFN-β*, *ISG56*, and *ISG15* were examined in uninfected BO (mock), infected BO (control), and infected BO treated with 10 µM camostat (camostat). (**d**) BO were infected with SARS-CoV-2 (1.3×10^{5} TCID50/well)

for 24 or 48 h, and then cultured with differentiation medium for 5 days. The amount of infectious virus in the supernatant was measured using the TCID50 assay. Statistical significance was evaluated by one-way analysis of variance (ANOVA) followed by Tukey's post hoc tests (*$p<0.05$). In **Supplementary Figures 2c-2d**, the data represent the mean ± SD from three independent experiments.

Supplementary Figure 3   RNA-seq analysis of infected BO

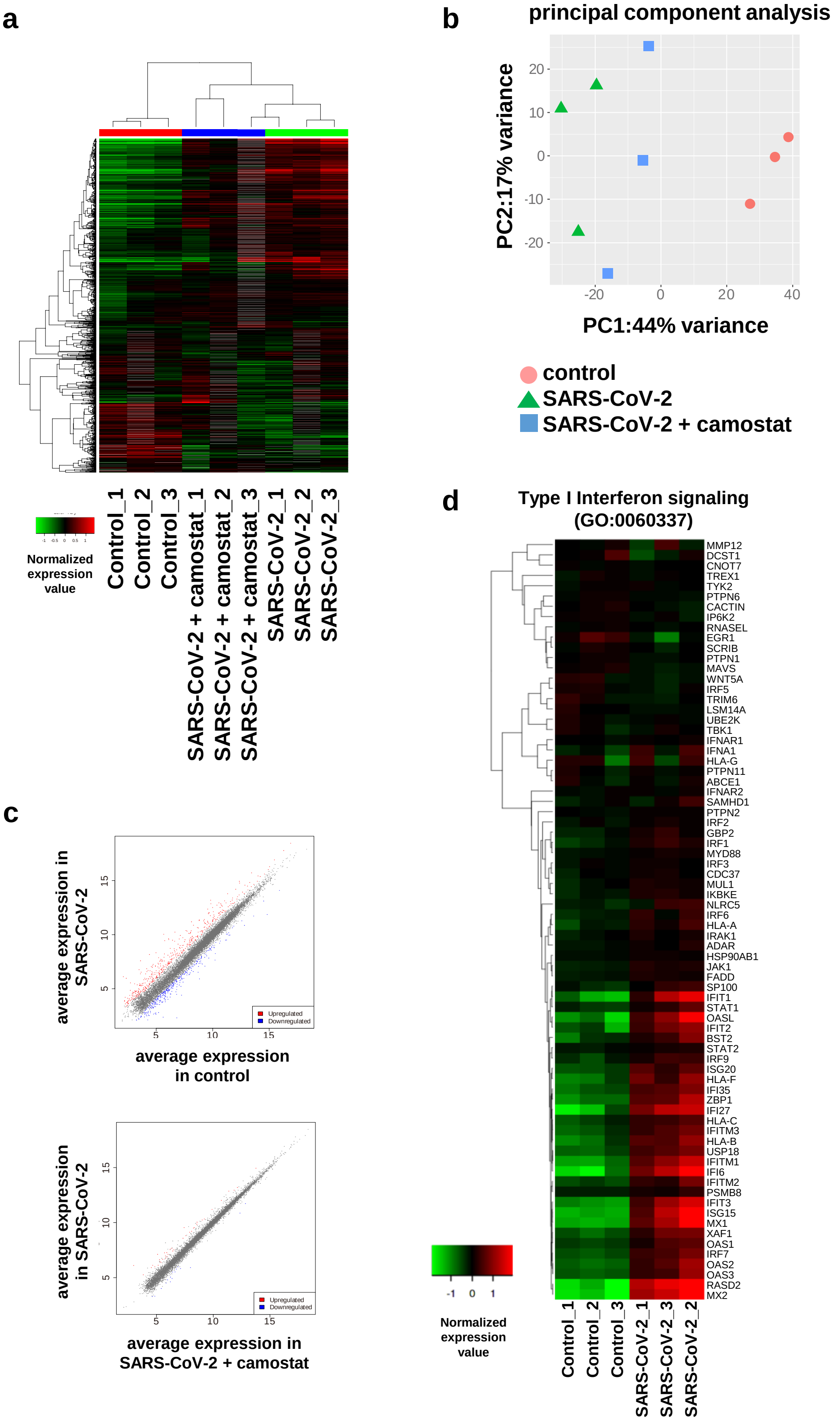

RNA seq analysis of uninfected BO (control), infected BO (SARS-CoV-2), and infected BO treated with camostat (SARS-CoV-2 + camostat). (**a**) A clustering analysis of 2,000 variable genes was performed. (**b**) Principal component analysis (PCA) of control, SARS-CoV-2, and SARS-CoV2 + camostat. (**c**) A scatter plot of control, SARS-CoV-2, and SARS-CoV2 + camostat. (**d**) A heat map of type I IFN signaling-related genes in control and SARS-CoV-2 is shown. Three technical repeats were performed per sample in the RNA-seq analysis.

Supplementary Figure 4 SARS-CoV-2 infects ciliated cells but not basal cells depending on ACE2 expression

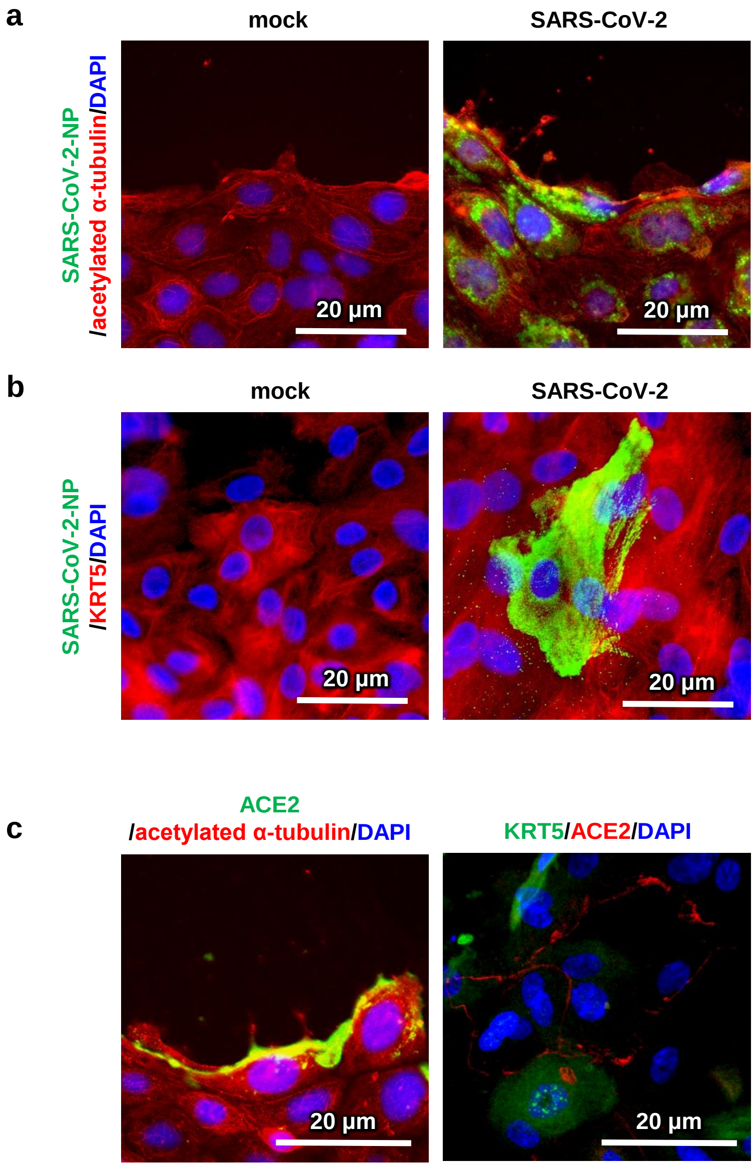

Suspended BO were infected with SARS-CoV-2 (1.3×10^5 TCID50/well) and then cultured with differentiation medium for 2 days. (**a**) Immunofluorescence analysis of SARS-CoV-2 NP (green) and acetylated α-tubulin (red) in suspended BO 2 days after the infection. Nuclei were counterstained with DAPI (blue). (**b**) Immunofluorescence analysis of SARS-CoV-2 NP (green) and KRT5 (red) in suspended BO 2 days after the infection. Nuclei were counterstained with DAPI (blue). (**c**) Immunofluorescence analysis of ACE2 (green) and acetylated α-tubulin (red) in suspended BO. Nuclei were counterstained with DAPI (blue). Immunofluorescence analysis of ACE2 (red) and

KRT5 (green) in suspended BO. Nuclei were counterstained with DAPI (blue). Immunofluorescence images are representative of three independent experiments.

Supplementary Figure 5 Gene expression analysis of BO and BO-ALI

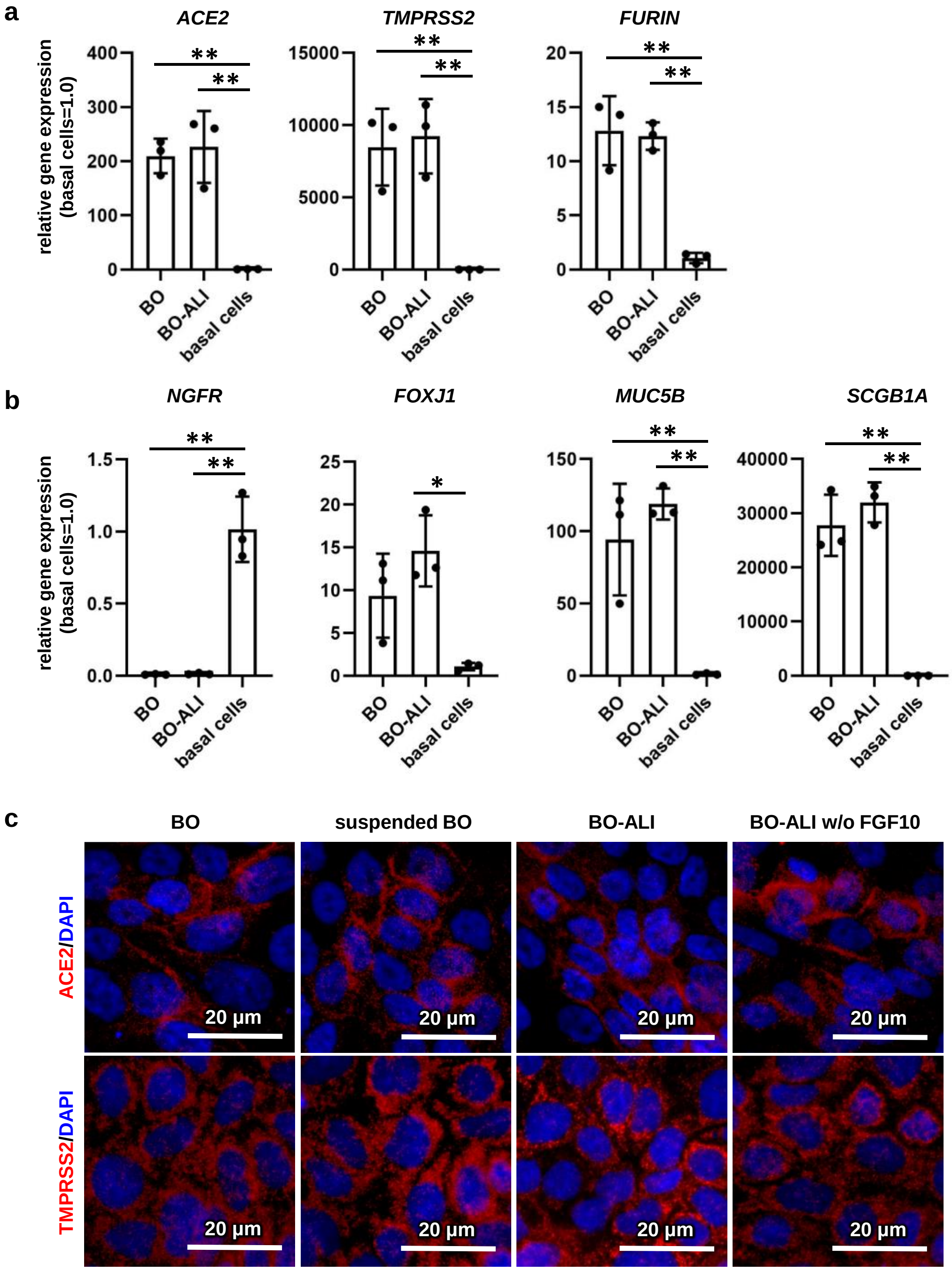

(**a**) The gene expression levels of *ACE2*, *TMPRSS2*, and *FURIN* in BO, BO-ALI, and bronchial basal stem cells (basal cells) were examined. (**b**) The gene expression levels of *NGFR* (basal cell marker), *FOXJ1* (ciliated cell marker), *MUC5B* (goblet cell marker), and *SCGB1A* (club cell marker) in BO, BO-ALI, and bronchial basal stem cells (basal cells) were examined. Statistical significance was evaluated by one-way ANOVA followed by Tukey's post hoc tests (*p<0.05, **p<0.01). Data represent mean ± SD

from three independent experiments. (**c**) Immunofluorescence analysis of ACE2 (red) and TMPRSS2 (red) in BO, suspended BO, BO-ALI, and BI-ALI w/o FGF10. Nuclei were counterstained with DAPI (blue). Immunofluorescence images are representative of three independent experiments.

Supplementary Figure 6 Temporal analysis of infected BO-ALI

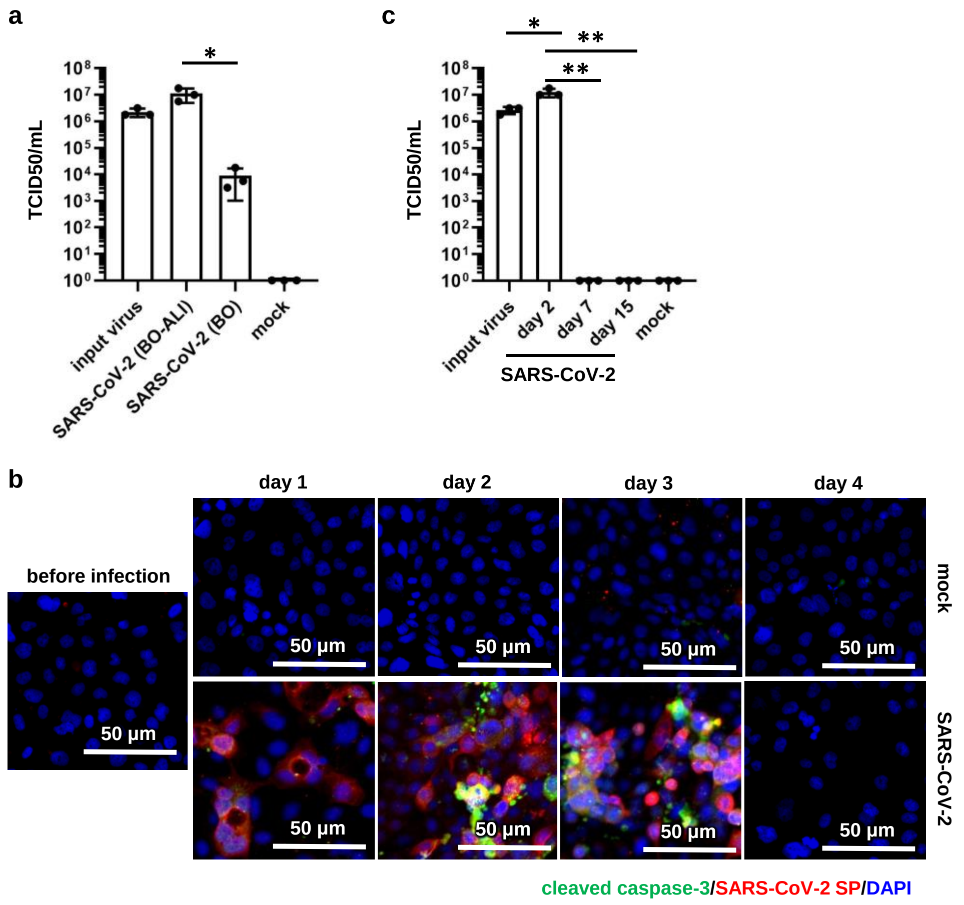

(**a**) BO and BO-ALI were infected with SARS-CoV-2 B.1.1.214 (1.3×10^5 TCID50/well) and then cultured with differentiation medium for 2 days. The amount of infectious virus in the supernatant of infected BO or BO-ALI was measured using the TCID50 assay. (**b**) BO-ALI were infected with SARS-CoV-2 (1.3×10^5 TCID50/well) and then cultured with differentiation medium for 1, 2, 3, and 4 days. Immunofluorescence analysis of SARS-CoV-2 NP (red) and cleaved caspase-3 (green) in the infected BO-ALI. Nuclei were counterstained with DAPI (blue). Immunofluorescence images are representative of three independent experiments. (**c**) BO-ALI were infected with SARS-CoV-2 (1.3×10^5 TCID50/well) and then cultured with differentiation medium for 2, 7, and 15 days. The amount of infectious virus in the supernatant of infected BO-ALI was measured using the TCID50 assay. Statistical significance was evaluated by one-way analysis of variance (ANOVA) followed by Tukey's post hoc tests (*$p<0.05$). Quantitative data represent the mean ± SD from three independent experiments.

Supplementary Figure 7 Gene expression analysis of innate immune response markers in infected BO-ALI

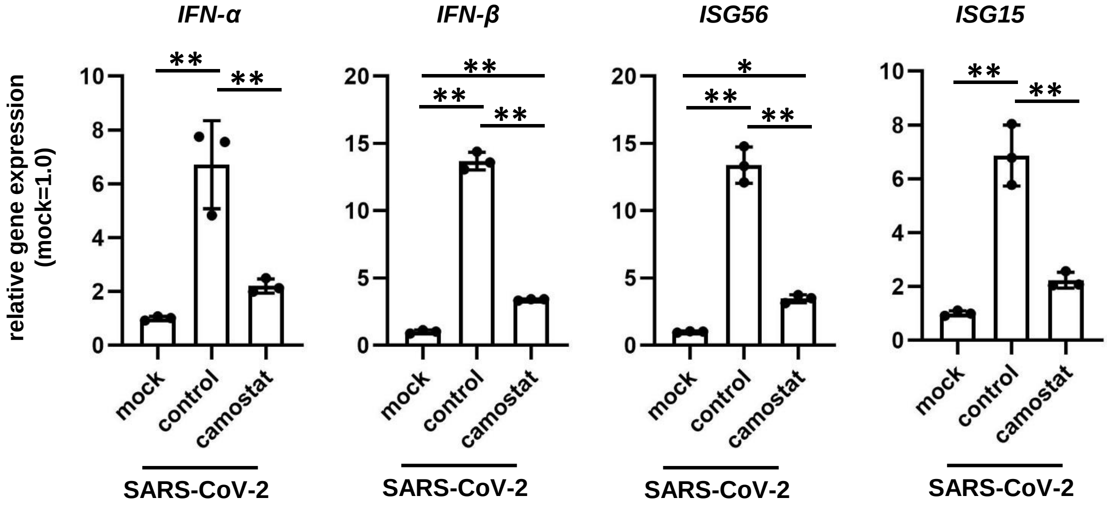

BO-ALI were infected with SARS-CoV-2 (1.3×10^{5} TCID50/well) in the presence or absence of 10 μM camostat and then cultured with differentiation medium for 2 days. The gene expression levels of *IFN-α*, *IFN-β*, *ISG56*, and *ISG15* were examined in uninfected BO-ALI (mock), infected BO-ALI (control), and infected BO-ALI treated with 10 μM camostat (camostat). Statistical significance was evaluated by one-way analysis of variance (ANOVA) followed by Tukey's post hoc tests (*$p<0.05$, **$p<0.01$). Data represent the mean ± SD from three independent experiments.

Supplementary Figure 8 Immunofluorescence analysis of BO-ALI cultured with medium with or without FGF

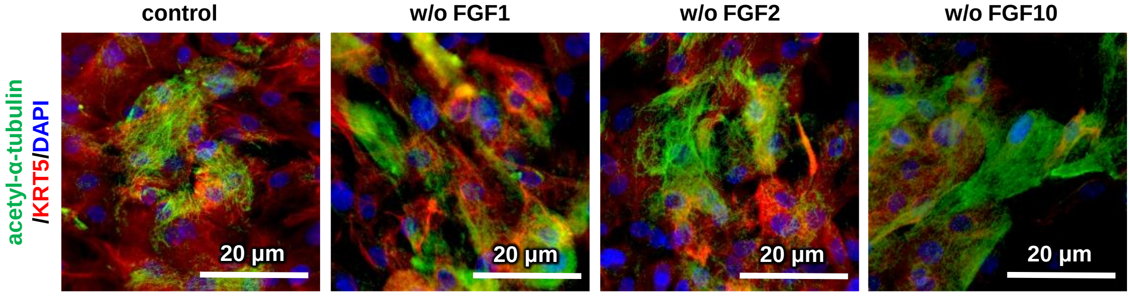

BO-ALI were cultured with differentiation medium with or without FGF1, 2, or 10. Immunofluorescence analysis of acetylated α-tubulin (green) and KRT5 (red). Nuclei were counterstained with DAPI (blue). Immunofluorescence images are representative of three independent experiments.

Supplementary Tables

Supplementary Table 1 Composition of expansion and differentiation media for BO

Composition (concentration)	Expansion medium	Differentiation medium
Advanced DMEM/F12	+	+
FGF2 (5 ng/ml)	+	+
FGF7 (20 ng/ml)	+	+
FGF10 (100 ng/ml)	+	+
Noggin (100 ng/ml)	+	−
R-spondin 1 (300 ng/ml)	+	−
Y-27632 (10 µM)	+	+
SB202190 (500 nM)	+	−
A83-01 (1 µM)	−	+
B27 supplement (1×)	+	+
N-Acetylcysteine (1.25 mM)	+	+
Nicotinamide (5 mM)	+	+
GlutaMAX (1×)	+	+
HEPES (10 mM)	+	+
Penicillin-Streptomycin (100 U/ml)	+	+
Primocin (50µg)	+	+

Supplementary Table 2 Primer list

target gene	Fwd primer	Rev primer
ACE2	ACAGTCCACACTTGCCCAAAT	TGAGAGCACTGAAGACCCATT
FOXJ1	GCCTCCCTACTCGTATGCCA	GCCGACAGGGTGATCTTGG
GAPDH	GGTGGTCTCCTCTGACTTCAACA	GTGGTCGTTGAGGGCAATG
IFN-α	GCAGATCACCCAGAAGATCG	GGCCCTTGTTATTCCTCACC
IFN-β	CCTTGCTGAAGTGTGGAGGA	CCAGGCGATAGGCAGAGA
ISG15	GCAGATCACCCAGAAGATCG	GGCCCTTGTTATTCCTCACC
ISG56	CCTTGCTGAAGTGTGGAGGA	CCAGGCGATAGGCAGAGA
MUC5B	GCCTACGAGGACTTCAACGTC	CCTTGATGACAACACGGGTGA
NGFR	CCTACGGCTACTACCAGGATG	CACACGGTGTTCTGCTTGT
SCGB1A	TTCAGCGTGTCATCGAAACCC	ACAGTGAGCTTTGGGCTATTTTT
TMPRSS2	GTCCCCACTGTCTACGAGGT	CAGACGACGGGGTTGGAAG

Supplementary Table 3 Antibody list

antigen	catalog number	company	antibody dilution ratio
ACE2	21115-1-AP	Proteintech	1/500 (IHC), 1/100 (IF)
acetylated α tubulin	sc-23950	Santa Cruz Biotechnology	1/200 (IHC), 1/100 (IF)
active caspase-3	G748A	Promega	1/250 (IF)
CC10	sc-365992	Santa Cruz Biotechnology	1/200 (IHC), 1/100 (IF)
cytokeratin 5	sc-32721	Santa Cruz Biotechnology	1/200 (IHC)
keratin 5	905504	Bio Legend	1/500 (IF)
mucin 5AC	sc-21701	Santa Cruz Biotechnology	1/200 (IHC)
SARS-CoV-2 NP	A2061	BIO Vision	1/400 (IF)
SARS-CoV-2 SP	GTX135356	GeneTex	1/200 (IF)
SARS-CoV-2 SP	GTX632604	GeneTex	1/500 (IHC)
