# Supplementary material for: Cell response analysis in SARS-CoV-2 infected bronchial organoids
Source: Commun Biol. 2022 May 30;5:516. doi: 10.1038/s42003-022-03499-2 (PMC9151746; doi:10.1038/s42003-022-03499-2)
Supplement: Supplementary file 2 — Description of Additional Supplementary Files [file 42003_2022_3499_MOESM2_ESM.pdf]

Description of Additional Supplementary Files

File name: Supplementary Data 1
Description: Source data underlying the graphs and charts.
