# Supplementary material for: Cell response analysis in SARS-CoV-2 infected bronchial organoids
Source: Commun Biol. 2022 May 30;5:516. doi: 10.1038/s42003-022-03499-2 (PMC9151746; doi:10.1038/s42003-022-03499-2)
Supplement: Supplementary file 4 — Reporting Summary [file 42003_2022_3499_MOESM4_ESM.pdf]

nature portfolio

Corresponding author(s): Kazuo Takayama
Toru Okamoto

Last updated by author(s): May 12, 2022



# Reporting Summary

Nature Portfolio wishes to improve the reproducibility of the work that we publish. This form provides structure for consistency and transparency in reporting. For further information on Nature Portfolio policies, see our Editorial Policies and the Editorial Policy Checklist.

## Statistics

For all statistical analyses, confirm that the following items are present in the figure legend, table legend, main text, or Methods section.

| n/a | Confirmed | |
|---|---|---|
| ☐ | ☒ | The exact sample size ($n$) for each experimental group/condition, given as a discrete number and unit of measurement |
| ☐ | ☒ | A statement on whether measurements were taken from distinct samples or whether the same sample was measured repeatedly |
| ☐ | ☐ | The statistical test(s) used AND whether they are one- or two-sided<br>*Only common tests should be described solely by name; describe more complex techniques in the Methods section.* |
| ☒ | ☐ | A description of all covariates tested |
| ☒ | ☐ | A description of any assumptions or corrections, such as tests of normality and adjustment for multiple comparisons |
| ☐ | ☒ | A full description of the statistical parameters including central tendency (e.g. means) or other basic estimates (e.g. regression coefficient) AND variation (e.g. standard deviation) or associated estimates of uncertainty (e.g. confidence intervals) |
| ☐ | ☒ | For null hypothesis testing, the test statistic (e.g. $F$, $t$, $r$) with confidence intervals, effect sizes, degrees of freedom and $P$ value noted<br>*Give P values as exact values whenever suitable.* |
| ☒ | ☐ | For Bayesian analysis, information on the choice of priors and Markov chain Monte Carlo settings |
| ☒ | ☐ | For hierarchical and complex designs, identification of the appropriate level for tests and full reporting of outcomes |
| ☒ | ☐ | Estimates of effect sizes (e.g. Cohen's $d$, Pearson's $r$), indicating how they were calculated |

*Our web collection on statistics for biologists contains articles on many of the points above.*

## Software and code

Policy information about availability of computer code

| | |
|---|---|
| Data collection | RNA sequencing was performed on an Illumina NextSeq500 or NovaSeq6000. Quantitative PCR was performed using StepOnePlus real-time PCR system. |
| Data analysis | Microsoft Excel and Graph Pad Prism 7 were used. RNA integrity was assessed with a 2100 Bioanalyzer (Agilent Technologies). Library preparation was performed using a NEBNext Ultra II Directional RNA Library Prep Kit for Illumina (NEB) or a TruSeq stranded mRNA sample prep kit (Illumina) according to the manufacturer's instructions. Sequencing was performed on an Illumina NextSeq500 or NovaSeq6000 platform in 152- or 101-base single-end mode, respectively. Fastq files were generated using bcl2fastq2. Adapter sequences were trimmed from the raw reads by cutadapt ver 2.7. The trimmed reads were mapped to the human reference genome sequences (hg19) using HISAT2 ver 2.1.0. The raw counts were calculated using featureCounts ver 2.0.0 and used for heatmap visualization with integrated differential expression and pathway analysis (iDEP, (http://ge-lab.org/idep/)). |

For manuscripts utilizing custom algorithms or software that are central to the research but not yet described in published literature, software must be made available to editors and reviewers. We strongly encourage code deposition in a community repository (e.g. GitHub). See the Nature Portfolio guidelines for submitting code & software for further information.

## Data

Policy information about availability of data

All manuscripts must include a data availability statement. This statement should provide the following information, where applicable:

- Accession codes, unique identifiers, or web links for publicly available datasets
- A description of any restrictions on data availability
- For clinical datasets or third party data, please ensure that the statement adheres to our policy

RNA-seq data can be obtained from GEO (accession number GSE150819). All other data can be disclosed upon a request.

# Field-specific reporting

Please select the one below that is the best fit for your research. If you are not sure, read the appropriate sections before making your selection.

☒ Life sciences ☐ Behavioural & social sciences ☐ Ecological, evolutionary & environmental sciences

For a reference copy of the document with all sections, see nature.com/documents/nr-reporting-summary-flat.pdf

# Life sciences study design

All studies must disclose on these points even when the disclosure is negative.

| | |
|---|---|
| Sample size | Data shown are representative of multiple independent experiments or biological triplicate experiments as described in figure legends. |
| Data exclusions | No data was excluded. |
| Replication | Biological duplicate or triplicate experiments were done with distinct aliquots of cells at intervals ranging from weeks between experiments and performed by two or three different researchers. All findings have been replicated. |
| Randomization | Not relevant to these experiments. |
| Blinding | Not relevant to these experiments. |

# Reporting for specific materials, systems and methods

We require information from authors about some types of materials, experimental systems and methods used in many studies. Here, indicate whether each material, system or method listed is relevant to your study. If you are not sure if a list item applies to your research, read the appropriate section before selecting a response.

### Materials & experimental systems

| n/a | Involved in the study | |
|---|---|---|
| ☐ | ☒ | Antibodies |
| ☐ | ☒ | Eukaryotic cell lines |
| ☒ | ☐ | Palaeontology and archaeology |
| ☒ | ☐ | Animals and other organisms |
| ☒ | ☐ | Human research participants |
| ☒ | ☐ | Clinical data |
| ☒ | ☐ | Dual use research of concern |

### Methods

| n/a | Involved in the study | |
|---|---|---|
| ☒ | ☐ | ChIP-seq |
| ☒ | ☐ | Flow cytometry |
| ☒ | ☐ | MRI-based neuroimaging |

## Antibodies

Antibodies used

ACE2 (PGI-21115-1-AP-150, Proteintech)
acetylated α tubulin (6-11B-1) (sc-23950, Santa Cruz Biotechnology)
active caspase-3 (G748A, Promega)
CC10 (E-11) (sc-365992, Santa Cruz Biotechnology)
cytokeratin 5 (RCK103) (sc-32721, Santa Cruz Biotechnology)
keratin 5 (905504, BioLegend)
mucin 5AC (45M1) (sc-21701, Santa Cruz Biotechnology)
SARS-CoV-2 NP (A2061, BioVision)
SARS-CoV/SARS-CoV-2 (COVID-19) spike protein (1A9) (GTX632604, GeneTex)
SARS-CoV-2 (COVID-19) Spike (GTX135356, GeneTex)
TMPRSS2 (H-4) (sc-515727, Santa Cruz Biotechnology)

| | |
|---|---|
| Validation | Information on validation of primary antibodies can be obtained from the URL below.<br><br>ACE2 (https://www.ptglab.com/Products/Pictures/pdf/21115-1-AP.pdf)<br>acetylated α tubulin (6-11B-1) (https://datasheets.scbt.com/sc-23950.pdf)<br>active caspase-3 (https://www.labome.com/product/Promega/G7481.html)<br>CC10 (E-11) (https://datasheets.scbt.com/sc-365992.pdf)<br>cytokeratin 5 (RCK103) (https://datasheets.scbt.com/sc-32721.pdf)<br>keratin 5 (https://www.biolegend.com/ja-jp/products/purified-anti-keratin-5-antibody-13378)<br>mucin 5AC (45M1) (https://datasheets.scbt.com/sc-21701.pdf)<br>SARS-CoV-2 NP (A2061, https://www.biovision.com/anti-ncovid-19-antibody.html)<br>SARS-CoV/SARS-CoV-2 (COVID-19) spike protein (1A9) (GTX632604, https://www.genetex.com/Product/Detail/SARS-CoV-SARS-CoV-2-COVID-19-spike-antibody-1A9/GTX632604)<br>SARS-CoV-2 (COVID-19) Spike (GTX135356, https://www.genetex.com/Product/Detail/SARS-CoV-2-COVID-19-Spike-antibody/GTX135356)<br>TMPRSS2 (H-4) (https://datasheets.scbt.com/sc-515727.pdf) |

## Eukaryotic cell lines

Policy information about cell lines

| | |
|---|---|
| Cell line source(s) | VeroE6/TMPRSS2 cells (JCRB1819, JCRB Cell Bank)<br>Normal human bronchial epithelial cells (hBEpC, Lonza) |
| Authentication | Cells were authenticated by the supplier performing STR analysis. |
| Mycoplasma contamination | VeroE6/TMPRSS2 and normal human bronchial epithelial cells were negative for mycoplasma. |
| Commonly misidentified lines<br>(See ICLAC register) | No commonly misidentified cell lines were used. |
